# Xylol the New Remedy for Small Pox

**Published:** 1872-04

**Authors:** C. R. C. Tichborne


### ARTICLE XI.

### *Xylol, the New Remedy for Small Pox.*

BY C. R. C. TICHBORNE, F.C.S., M.R.I.A.

Xylol, xylene, or ethyl-benzine, as it has been respectively called, is one of a homologous series of hydrocarbons, of which the well known benzine and toluene form the two first. These hydrocarbons, are all formed from coal-tar-naptha. Xylol was first procured by Hugo Muller, but its nitro-compound had previously been discovered by Warren De la Rue, in 1856. Coal-tar-naphtha is submitted to fractional distillation until the part which boils at 141° is separated ; this is submitted to the action of fuming sulphuric acid, which dissolves the xylol and leaves the other hydrocarbons. The xylol is then separated by distillation from this mixture.

Xylol is said to have been used by Dr. Zuelzer, the Senior Physician at the Charité Hospital at Berlin, with great success in cases of small-pox. The theory of its action would appear to be that xylol is taken up by the blood, and acts as a disinfectant. The vapor seems to the writer to possess faint and not very well marked, anæsthetic properties ; this may be due to the presence of a small quantity of benzol, or the other hydrocarbons. The antispeptic properties of this group of compounds are well known, and thus, probably, the specific action of this one. The boiling point is variously stated at 139° to 140°. The specimens examined by the writer generally commenced to boil at about 135° C the speeific gravity was ·866.

It is said that the purity of xylol is of importance, but unfortunately there is no very ready method by which the ordinary practitioner might detect its purity. It should be soluble in fuming sulphuric acid, but it is not soluble in the ordinary sulphuric acid of the Pharmacopœia.

It has a faint odor something like benzol, and an aromatic taste. The doses are three to five drops for children; ten to fifteen drops for adults, every hour to every three hours. It is quite harmless in reasonable doses. In Berlin it is given in capsules. As it is very insoluble the best method of giving it would be in an emulsion of almonds. When once assimilated it is rapidly oxidized in the body, this fact being demonstrated by the production of a peculiar odor in the urine, which, however, is quite distinct from xylol itself.—*Med. Press and Circular.*

---

